# The Transactions of the Provincial Medical and Surgical Association, Vol. X.

**Published:** 1842-07-01

**Authors:** 


### Medico-Chirurgical Review,

### No. LXXIII.

[No. 33 of a Decennial Series.]

APRIL 1, to JULY 1, 1842.

### The Transactions of the Provincial Medical and Surgical Association. Vol. X. Churchill, 1842.

The volume before us is inferior in size, at all events, if not in interest, to most of its predecessors. Its contents are—1. The Retrospective Address delivered at the Ninth Anniversary Meeting of the Provincial Medical and Surgical Association, held at York, August 4th and 5th, 1841. By Robert J. N. Streeten, M.D.—II. On the Medical Topography of Exeter and the Neighbourhood, being a Sketch of the Geology, Climate, Natural Productions,.and Statistics of that District. By Thomas Shapter, M.D.—III. Observations on the Climate of Herefordshire compared with that of the Neighbourhood of London, with Meteorological Tables, &c. By Lieut.-Colonel Philip Yorke.—IV. Medical Topography of Shrewsbury and its Neighbourhood. By T. Ogier Ward, M.D.—V. Case of a Pin passing from the Appendix Vermiformis into the Bladder. By William Dashwood Kingdon, M.D.—VI. Case of Tumour developed in the midst of the Cauda Equina. By W. W. Fisher, M.D.—VII. Further Observations on the Variolæ Vaccinæ. By Robert Ceely, Esq.—VIII. Report of Cases at the Chester General Infirmary during the Years 1838, 1839, and 1840. By Thomas Beavill Peacock, Esq.

#### The Retrospective Address.

This has been compiled with care, and is executed with discrimination. We shall select one or two points from it.

*Dr. Foville's latest Researches on the Brain.*—The inferences drawn by Dr. Foville from his later researches are, first, that the fibrous parts of the brain are conductors, some from without to within, others from within to without; that these conducting parts may be distinguished into afferentes and efferentes; that the distinct course of both the one and the other may be demonstrated; that the first are inserted especially into the circumference of the gray substance, and the second into its internal surface; that the afferent conductors are those fibres which are intermediate between the posterior parts of the spinal marrow, the optic and olfactory nerves, and the circumference of the convolutions, and that the efferent are those parts connecting the internal surface of the convolutions with the anterior pyramids: second, that the gray substance of the convolutions, intermediate between the two preceding orders of fibrous parts, seems to be the material substance through the instrumentality of which the will directs the movements of the body.

*Summary of Experiments on the Motions and Sounds of the Heart.*—Dr. Clendinning has drawn up a report of the Committees of the British Association for the Advancement of Science. The report contains the conclusions deducible from the two series of experiments and observations instituted by these Committees both on the motions and sounds of the heart. Among the most important of these in a practical point of view are, that the order of the motions of the auricles and ventricles is by continuous succession, rather than by alternation of action, the auricles contracting abruptly after the rest or pause, and the ventricles immediately after the auricles; that the normal systolic action of the auricles is energetic and almost instantaneous, and that the normal auricular diastole is gradual, continuous, and wholly passive; that the systole of the ventricles is gradual in its development; that the arterial diastole or pulse perceptibly succeeds to the cardiac systole; that the first sound of the heart depends partly on the abrupt closure of the aurico-ventricular valves, but is mainly owing to cardiac muscular tension alone; that the auricular systole is attended by an intrinsic sound resembling that of the ventricles, but more short, obtuse, and feeble, often difficult of detection, owing to its being absorbed in the louder ventricular systolic sound immediately succeeding; that the sounds of friction in pericarditis may, when well marked, be expected to be double, and sometimes triple, or more; that the sounds of the structurally healthy heart are liable to modifications by alterations in the condition of the fluids; and that the suction influence on the venous circulation, attributed to inspiration by various writers, is well founded.

*Inefficiency of Remedies for Fever.*—Some remarks on the treatment of continued fever have been made by Dr. Eager, of Manchester, and a comparison drawn between the effects of emollients, antiphlogistics, and purgatives. The conclusion at which Dr. Eager arrives is, that in the milder forms all kinds of treatment, when not too exciting, have proved more or less successful, and that, as in the more severe forms patients have recovered under the most opposite methods, it is probable the means adopted have had little influence, and that nature alone acted. It should perhaps be stated that many of Dr. Eager's cases were observed in Paris and the rest in Manchester. In all the fatal cases examined after death, one only excepted, the follicular affection was present.

*Scarlatina Encephalica.*—An essay of considerable practical importance on the Nature and Treatment of Scarlet Fever connected with Cerebral Symptoms has been contributed by Dr. T. F. Cornell. In the cases to which the observations of the author refer, and which he proposes to term scarlatina encephalica, depletive measures, notwithstanding an apparent imperative demand for them on account of the severity of the head symptoms, seem to have proved fatally injurious, while under a contrary mode of treatment, the exhibition of wine and other stimulants, many of the cases were conducted to a favorable termination.

*Application for "Pitting" in Small-Pox.*—The application of mercurial plaster in variola to the face and other parts, with the view of preventing the formation of the pustules, has been successful in many cases treated by M. Chomel and others. A paper on this subject has been read by Dr. J. F. Olliffe before the Medical Society of Paris. The plasters should be applied while the eruption is in the papular stage; the formation of pustules is thereby prevented and the progress of the eruption checked, the formation of cicatrices does not take place, and the disease is stated to be conducted to a safe termination. The action of the mercurial is thought by Dr. Olliffe to be specific. Sulphur ointment has been proposed as a substitute for this remedy by Dr. Midaveine, of Ghent, and is stated to have proved equally efficacious. The last observation is, we dare say, true enough. Probably any grease would do as well as the sulphur ointment or mercurial plaster.

*Diagnosis of Hydrops Pericardii.*—Mr. Mackenzie has pointed out that, in cases where hydrops pericardii is combined with co-existing effusion into the pleura, the patients are unable to bear the horizontal position, but that where the fluid is confined to the pericardium the horizontal position is preferred, probably from the weight being thus taken from off the diaphragm.

*Diagnosis of Nervous Palpitation.*—The nervous palpitation of young females often simulates patency of the aorta. Dr. Corrigan points out as diagnostic marks that in the permanent patency of the aorta "*the bruit in the carotid is synchronous with the diastole of the arteries;* whereas in the hysteric or chlorotic affection "*it is not confined to the duration of the diastole of the artery, and is very frequently a continuous, uninterrupted, long, rumbling sound.*"

We have misgivings on both these last points of diagnosis.

*Aneurysm of the Pulmonary Artery.*—This was a case related by Mr. Fearn, of Derby, in which, on examination, after fatal hæmoptysis, "a distinctly defined *aneurysmal sac,* as large as a nutmeg," was observed jutting into a tuberculous excavation on the upper lobe of the left lung. "The parietes of the sac were thin, and it did not contain any fibrinous layers; a vessel, the size of a small crow-quill, leading from a considerable trunk of the pulmonary artery, was distinctly traceable into the sac."

*Detection of Sugar and Albumen in the Urine.*—An ingenious and simple apparatus has been devised by M. Biot for detecting the presence of sugar in diabetic urine upon optical principles. The method consists in ascertaining the action of the fluid suspected to contain sugar on polarized light, and we are assured that in this manner the smallest quantity of sugar may be instantly detected in the urine of a diabetic patient, and the progress of the disease and the effect of treatment thus ascertained with the utmost facility. The same principle may be applied also to the detection of albumen, the action on the polarized ray being in this case in a contrary direction, and M. Donné proposes also to determine by the instrument of M. Biot the presence of animal matters, not albuminous, in the urine from the negative results afforded.

*Urine a Test of Pregnancy, Chlorosis, and Phthisis.*—The urine has been examined in pregnancy and in various states of disease by M. Donné, who has been able to determine, from the microscopic character of the salts deposited, with great probability, if not with absolute certainty, the existence of pregnancy. The urine in chlorosis he found to be destitute of all traces of iron, although in health a certain quantity is always present; and in phthisis also the urine presents some peculiar qualities which are considered by M. Donné so characteristic as to afford a means of diagnosis in doubtful cases.

Very like a whale.

*Injections into the Uterus.*—A warm discussion has been carried on in France, respecting the danger to be apprehended from injections into the uterus in various diseases of that organ. M. Hourmann found that an injection thrown up into the uterine cavity in a case of leucorrhœa, produced immediate pain in the left iliac region, and was followed by symptoms of metro-peritonitis, for which the most energetic treatment was required. From experiments afterwards performed, it was ascertained that injections with an arterial syringe might be made to pass into the fallopian tubes. Similar occurrences have also been reported by M. Leroy d' Etiolles, and M. Guillemin, of Rombas. The subject has been also investigated by M. Vidal de Cassis, and M. Leroy d' Etiolles; and discussions have taken place upon it in the Medical Society of Paris.

We cannot but think these injections hazardous.

*Effects of Iodine.*—A paper on the circumstances which favour the therapeutic action of iodine has been drawn up by Dr. Mojsisovits, from the examination of upwards of eight hundred cases, in which the remedy was employed. The iodide of potassium and iodide and biniodide of mercury are considered to be the most useful preparations; the tincture of iodine the most uncertain and injurious. The activity of the remedy is said to be greatest in clear and dry weather, and when the epidemic constitution is inflammatory and catarrhal. On the other hand, when small-pox, puerperal fever, and diarrhœa are prevalent, its action is almost null. Bread, potatoes, rice, oatmeal, and all substances which contain fecula or starch, are said to be incompatible. Iodine causes as critical effects, salivation and a miliary eruption or rash resembling scarlatina. Salt-water baths contribute much to favour the action of the iodine.

All we can say is, that if bread, potatoes and gruel can neutralize the action of iodine, there is plenty of it wasted in this country. In spite of these dangerous aliments, we have seen it of service. But to proceed.

The diseases in which it was found most efficacious were certain chronic syphilitic affections, and especially such as are the combined result of syphilis and mercury, and various scrofulous degenerations. The injurious effects sometimes arising from the employment of the iodide of potassium and the iodide of starch have been pointed out by Dr. Adair Lawrie, of Glasgow. The mucous membranes of the eyes and air-passages are said to be especially liable to become affected. In one instance the employment of the medicine was followed by urgent dyspnœa and loss of voice; in another, by excruciating head-ache, acute pain in the eyes, profuse secretion of tears, and intense pain in the side, (nostrils ?) with swelling and continuous discharge of clear serous fluid; in a third, by fatal dyspnœa; in a fourth, by acute congestion of the conjunctiva, pain of chest, hoarseness, cough, and dyspnœa; in a fifth, by profuse papular eruption, which disappeared on the iodine being omitted, and re-appeared on its being again resumed, followed by sore throat, acute dyspnœa, and hoarseness, with fatal result, the mucous membrane of the upper part of the larynx, rima glottidis, and epiglottis being found œdematous on inspection; in a sixth, by intense headache, slight salivation, and sore throat; and in a seventh, by severe headache. Dr. Lawrie has never seen these preparations act as irritants to the mucous membrane of the intestinal tube, nor has he ever observed them to produce emaciation or atrophy of the mammæ or testicles.

Case of a Pin passing from the Appendix Vermiformis into the Bladder. By *William D. Kingdon*, M.D., Physician to the Exeter Dispensary.

J. P. aged 7. In the early part of January, 1836, he awoke in the night time, complaining of great difficulty of micturition, not being able to pass more than two or three drops of urine at a time. In this state he continued, suffering little pain, and that only from the retention of urine, for upwards of a week; when one morning, making greater efforts than usual, he perceived something of a whitish colour moving about at the orifice of the urethra, and taking hold of it, drew out a female worm (*ascaris lumbricoides*), which was followed by an immediate relief from the foregoing symptoms. No farther notice was taken of the circumstance until twelve or thirteen months afterwards, when the dysuria recurred, and lasted nine or ten days, at the expiration of which term he had severe pain at the neck of the bladder, and said that there was something crawling in his penis; on examination, his mother discovered another worm, and drew it out as in the former instance, and with the same relief. In six months afterwards the same symptoms returned, but subsided in a few days on the evacuation of another worm. On the 4th of October, 1838, he complained of pain in the perinæum and at the extremity of the penis, which continued night and day for more than a week, when it entirely subsided on the passage of another worm. He remained free from pain until January 11th, 1839, when the same distressing symptoms again recurred with aggravated severity, and lasted for two or three days: during this period he was unable to emit any urine, and it was for the first time thought necessary to call in the aid of a medical practitioner, who, on introducing the catheter, drew off a large quantity of water; in the course of the same afternoon a worm crept from the urethra, and, as before, the little sufferer experienced immediate relief. The pain however recurred much more frequently, and the urine was obliged to be drawn off repeatedly. The boy's appetite began to fail, and he lost flesh rapidly. Various professional gentlemen were consulted, but he received no benefit, and gradually became worse.

On the 8th of February, 1839, he came under Dr. Kingdon's care in the Exeter Dispensary. He then complained of occasional pain in the perinæum and at the extremity of the penis; to make use of his own words, "Like as if there was a worm there, wanting to poke his way out." There was at times difficulty in passing his urine, but not requiring the use of the catheter. Under the use of sedative medicines, he was much relieved, but, on the 12th April, the symptoms returned with more severity than ever. The catheter was introduced, and afforded instant relief; shortly afterwards a worm made its way through the urethra, as on a previous occasion. Up to this period the boy had always voided his urine through the natural passage; but subsequently *per anum*. The pain now recurred two or three times in the course of the day, but was alleviated as soon as he could evacuate the urine from the bladder; the pain was likewise lessened on pressing the perinæum with his hand; occasionally too a day would pass without any uneasy symptom. In the beginning of May, he was sounded, but no calculus was found. He became much worse, and frequently complained of the worm attempting to force its way out, and when in great pain a quantity of purulent matter oozed from the urethra. On the 20th of October he became blind; the pulse averaged 120; the countenance was anxious; the appetite for food very small, and that only for liquids. Very little urine was voided for the space of a fortnight, and his pain was more severe than ever; for this he took one-eighth of a grain of belladonna every five or six hours, and with considerable temporary relief. On the 24th his sight returned, and he became so much freer from pain that his medicines were omitted. On the 9th of November he was entirely free from pain, and any urine secreted was voided through the natural passage. Two worms were brought away by stool, and a third was found in the bed next morning. Still he became gradually weaker, and on the 15th, died.

*Examination of the Body.*—Emaciation. The whole intestinal canal discoloured and presenting traces of inflammatory action, but the colon and rectum much more so than the small intestines; mesenteric glands enlarged; the *appendix vermiformis*, instead of occupying its natural situation, had descended into the pelvis, and about an inch from its termination was firmly united to the superior and lateral portion of the bladder a little above the junction of the ureter with this organ; the bladder itself was smaller than natural, and firmly contracted at its lower part upon a hard substance, which, on laying open the cavity, proved to be a calculus of the triple phosphate form, measuring in length one inch and six-tenths, and in circumference two inches and nine-tenths; the parietes of the bladder were much thickened, and on laying them open about half an ounce of purulent matter escaped; the calculus was firmly pressed upon the internal orifice of the urethra, preventing almost entirely the flow of urine in that direction; the mucous coat of the bladder was ulcerated in two places, and on the mesial side of the opening of the right ureter, and a little above it, were two fistulous openings, the septum between the two being very slight, communicating with the interior of the *appendix vermiformis;* both ureters were much enlarged and inflamed, and both kidneys larger than natural, and so completely filled with pus that scarcely a healthy portion was discernible.

The calculus being carefully divided, displayed in its centre a large pin, which, as Dr. Kingdon justly remarks, satisfactorily accounts for the singular appearances above detailed. The poor boy must have swallowed the pin, which, after traversing the small intestines, formed a lodgment in the *appendix vermiformis;* here the irritation caused by it must have given rise to inflammation and adhesion of the process to the exterior of the bladder, and subsequently, by ulceration, to the passage of the pin into the urinary bladder, where it formed the nucleus of the calculus discovered after death, though not detected during life. The fistulous communication with the bladder will likewise very readily account for the voiding of the urine from the anus, the natural orifice being closed by the calculus; and also for the passage of the worms through the urethra on the several occasions mentioned.

A curious case.

### Case of Tumor developed in the midst of the Cauda Equina.

By *W. W. Fisher,* M.D. Downing Professor of Physic, Cambridge.

Taylor, a tailor, aged 38, intemperate, first seen, January 1840. In 1837, he injured, whilst riding, the lower part of the loins by the back part of the saddle, and from that period he began to suffer from pain in the lumbar and sacral regions, which was attributed to rheumatism; the pain gradually became more violent, and extended down the legs, which began to swell. He was obliged to give up work in June, 1839, and became bed-ridden in the September of the same year; he could not however lie down, but rested on his hands and knees. He was then unable to move either his loins or lower extremities; but he had the free use of neck, shoulders, and arms. The pain, which had formerly been chiefly confined to the region of the sacrum, was now more particularly felt across the seat, extending from one ischium to the other. There was great numbness throughout the lower extremities; and although no sensation was in the left leg or toes by touch, nevertheless he complained strongly of a feeling of heat in the parts. There was some degree of feeling, on touch, left in the right leg. The legs were very œdematous; there were large ulcerations on those parts of the knees on which he rested, yet he did not experience pain from them. He was generally sleepless, but did not suffer from head-ache; his breathing was easy, his pulse undisturbed, and his appetite good. He had difficulty in making water; and his bowels were generally confined, and at times so obstinately constipated as to resist the action of cathartics and purgative injections. An issue had been placed on the region of the sacrum, the discharge from which was thin; this was rendered of a more purulent character by the use of iron, from which he seemed to derive more benefit than from any other medicament, especially as regarded the making of water. He died in May.

*Examination.*—Back only inspected. The sacrum seemed more protuberant than usual; this appearance however arose from the loins being more depressed. The arches of the dorsal and lumbar vertebræ and the posterior wall of the sacrum were removed; the laminæ of the lumbar vertebræ, as well as their bodies, were partially affected with caries.

Viewed posteriorly, the dura mater appeared to be in its natural state until it reached the extremity of the spinal cord ; but from that point to the end of the sacrum it was wanting, so that the mass of tumors was exposed to view. The morbid growth extended more towards the left than the right side of that portion of the spinal canal in which it was situated. The spinal cord was cut across, about the middle of the back, and the inferior portion of it was removed; nearly the whole of the diseased mass came along with it. The cord appeared to be quite sound throughout. The diseased mass had a lobulated form, and was involved in the cauda equina; and although it was traversed by a few of the nerves, nevertheless the greater portion of the latter could be detached from it.

It was difficult to determine the seat of the tumor when examined posteriorly ; but anteriorly the dura mater was sound throughout; and the arachnoid membrane, especially at the upper portion of the tumor, could be traced intact between the latter and the dura mater. Here and there processes were observed to pass from the arachnoid to the diseased structure, but they were similar to those met with between the arachnoid and the pia mater in their natural state. The morbid growth presented several traces of vascularity in the centre, and had a scirrhous appearance. The upper portions of the tumor were softer, and were involved in a fine glistening covering; sections of several portions of them showed them to be composed of a grey, semi-transparent, jelly-like substance, infiltrated amidst reticulated tissue, and marked with sanguineous striæ, several of which appeared like true vessels.

Dr. Fisher thinks there can be little doubt that the disease was seated in the pia mater.

### MEDICAL TOPOGRAPHY OF EXETER.

Dr. STREETEN has communicated a very careful and valuable memoir upon this subject. We shall pick out an observation or a fact here and there that seems instructive.

*Types of Prevalent Disease.*—Within Dr. Streeten's observation the general aspect of disease has partaken of two very separate and distinct characters. During the few years immediately preceding 1828, affections of the serous membranes for the most part prevailed ; thus the cases chiefly met with were those of pleuritis, peritonitis, and, amongst children, hydrocephalus, all exhibiting a type of disease requiring the most prompt and persevering antiphlogistic treatment. Since that period, however, diseases having their origin in the mucous membranes, or else involving them in their course, have been of the most usual occurrence, and these have required a treatment rather mild than heroic; in fact it was early learned that the type of disease was changed, and the method of cure which had been previously requisite was now any thing but applicable.

We fancy that this remarkable tendency of late years, to affections of the mucous membranes has not been limited to Exeter. But to proceed: —In 1825, inflammations of the serous membranes were exceedingly prevalent; peritonitis, in its severest forms, was of the most frequent occurrence; and, amongst children during this year, hydrocephalus was so frequent as really to justify one in styling it an epidemic. During the autumn of 1829, English cholera in its most rapid and urgent forms occurred, and was attended by a very signal mortality. In 1831, there prevailed an influenza of a severe character. 1832 was conspicuous, during the months of July and August, for the prevalence of the malignant cholera. In 1834, there occurred a slighter influenza. In 1836, small-pox; followed, in 1837, by hooping cough, and then by scarlet fever; each of these infantile diseases during this period was very fatal in its consequences. In 1837, influenza again occurred; it was very general, and attended, as was the case throughout England, with a severe and painful series of symptoms; and in the spring of 1838, a peculiar form of spotted fever occurred.

*Fever.*—In Exeter, February is the month the most prone to fever, and August and September are the least so, as is also the case in a lesser degree with the three preceding and two following months; with regard to December it has elsewhere been pointed out why it may not be taken into account. We see therefore that the fine cold bracing weather, which people so usually congratulate themselves upon as being free from fever, is really the weather most liable to it; while the warm and sultry months of summer are those in which it prevails the least. It is not a little remarkable that the year 1827, in which the greatest proportion of fever cases occurred, was one in which the fewest number of deaths took place; while in 1833 and 1834, years in which the proportion of fever was but small, the amount of deaths was above the average. The same observation also holds good, as regards the months; for August and September, which, of all the months are the most fatal, are seen to be the most free from fever; while February, the least fatal, is the most prone to its attacks.

We confess that we are staggered by the assertion, that August and September are the most fatal months. This is not the case elsewhere; and indeed it is irreconcileable with another statement of Dr. Streeten's, that " September is by far the healthiest month of the whole year."

*Complications of Fever.*—It appears to Dr. S. that, in Exeter, the most frequent complication is with affection of the mucous membranes of the stomach and bowels, then with the viscera of the chest, and more rarely with the cerebrum. In the other and more simple fevers of the district, the chest is the organ most frequently attacked, then the abdomen, and then the brain. But, in placing cerebral complication so low, he refers to the early stages of fever, and not to any symptomatic disorder of the brain which may supervene towards the fatal close. When the brain is primarily affected, it is generally by congestion, accompanied by low muttering delirium, with coherence when roused; active and violent delirium is comparatively rare. Primary affection of the cerebrum rarely assumes an epidemic character; it occurs more usually in a few cases only, and at the same time that other cases occur which are characterised by difficult and more general complications. When cerebral affection occurs it may however for the most part be observed that not only is it complicated with lesion of other organs, but that most usually the presence of these lesions had been previously manifested. Affection of the chest, as already observed, most frequently occurs in the ordinary simple fevers; it is in fact very rarely that cases of synochus, if not complicated with the more urgent derangements of the chest, are not attended by some short irritating dry cough, which after a day or two passes into a moist one.

Fever, with pulmonic complication, occurs chiefly during the Winter and Spring.

In 1839 and 1840, fever assumed a hæmorrhagic tendency, and many died in consequence of the bloody discharges poured from the bowels, and which no means appeared capable of restraining.

During the last ten years it has occurred twice if not three times that the external mucous surfaces in children, during a scarcely appreciable attack of fever, have shown a great liability to take on an inflammation of a bad character, attended by copious muco-puriform discharges. In 1834, this was peculiarly the case with the female organs of generation; so much so, that if seen without a knowledge of the presence of fever and its epidemic character, suspicion might have been raised that disease had been communicated, attended with violence.

In 1841, there prevailed amongst children a low fever, accompanied with diarrhœa, in which not only the throat, (from which in some cases false membranes have been thrown off,) but the conjunctiva has become inflamed. The eyelids, generally swollen, have with difficulty permitted the state of the ball to be observed; soon the secretion becomes copious, and so tenacious as to almost preclude the possibility of opening the eye. In this way the acrid discharge is pent up; and the little sufferer, in case of life being spared, recovers, with the eye materially injured, if not entirely destroyed.

*Scarlet Fever*.—In 1838, it assumed a peculiarly bad aspect. Though apparently of an inflammatory character, it could bear but little depletion, and the use of purgatives appeared particularly hazardous; under such a course of treatment life seemed to vanish,—so silently and rapidly did the vital powers subside. It appeared, in fact, in the early commencement of this epidemic, that all attempts to counteract its influence were baffled, and that death would ensue in spite of every effort. Latterly Dr. Streeten adopted Dr. Peart's plan of giving ammonia. Under its influence the patients appeared to cool, and express immediate relief,—indeed subsidence of the more urgent symptoms quickly ensued.

Dr. S. mentions a case illustrative of the persistency of the contagion of scarlet fever.

" A young lady had this affection; two months after her most perfect recovery I was consulted as to the safety of her visiting some distant relatives where children were. The assurance was ventured of perfect safety. To my surprise, in two distant houses where these visits were paid, the scarlet fever broke out. There could be traced no other previous cases in either neighbourhood, so that the only conclusion to be arrived at was that the contagious principle had been retained and conveyed by this young lady. This occurred in the Autumn and Winter of 1840."

*Re-vaccination.*—Though, perhaps, we can scarcely go *quite* so far as Dr. Streeten, we are persuaded there is a great deal of truth in what he says:—" Re-vaccination is often resorted to; I have watched its progress in a great many cases; and where satisfied of a previously efficient vaccination, have *never* seen it go through a regular progress, or in any way present a vesicle from which lymph ought to be taken. The conviction therefore presses itself upon me that there is no other use in re-vaccination than as a test of the regularity of the previous vesicle. In this respect it appears to me to be eminently useful, and by no means to be neglected where the slightest doubt is entertained. From what I have seen of re-vaccination, I should, however, be inclined to place no confidence in it as a test unless there were present very sensible evidence of the specific virus being absorbed, as indicated by the formation of an irregular vesicle, attended by a certain degree of surrounding inflammation. It is too often the practice to insert from a glass into the arm a small quantity of the dried virus, and then, because no effect has followed, to proclaim that the patient resists the infection in consequence of the previous operation. Thus an imperfect attempt not only lulls into a false security, but, by its failure, throws undue discredit upon the protective power of vaccination."

*Diabetes Mellitus and Phthisis.*—" Cases both of diabetes mellitus and insipidus are by no means uncommon. Every case of true diabetes that has come under my observation here has terminated in tubercular consumption. This has been so uniformly the case, that the conviction forces itself upon me that it is essentially a symptom of scrofulous disease, and that the kidney is made to be an emunctory of these matters, otherwise colliquatively discharged by the skin."

*Scrofulous Glands.*—Dr. Streeten speaks highly of the ioduret of lead in these cases. But possibly, he is sanguine, for he thinks that true scirrhus of the mamma has been cured " in more than one instance" that he has witnessed by iodine.

*Epilepsy* has in many instances been much benefited, and in some few entirely relieved, by a sustained exhibition of the valerian and hydrocyanic acid, together with the daily use of some slight tonic aperient, as rhubarb combined with soda. The effects of this treatment have been very striking.

*Mercury and Phthisis.*—" Three cases of mental delusion, in connexion with consumption, and after free salivation by mercury, have occurred to my observation, with so much singular coincidence, that I am induced not only to refer to them here, but to style them phthisis, complicated with mercurial irritation. In each the patients presented the usual character of the incipient stage of phthisis; but superadded to this was an impression that their whole system was impregnated with mercury, which in two cases had been taken for syphilitic affection, and in the third for an accidental attack of swelled testicle. So strongly rooted was this impression that they maintained they smelled it in their perspirations, tasted it in their saliva, were convinced it was in their secretions, and that to this, and this only, were attributable the unpleasantness of the symptoms they were labouring under. This state of things in each occurred until the symptoms of phthisis became fully developed, which usually they did suddenly; then the delusion subsided, and the patient went through the ordinary course of a very rapid decline." 152.

We quote this passage because we feel assured that even a moderate mercurial course too frequently gives rise to phthisis pulmonalis. And we have seen the same sort of delusion, not carried, perhaps, to such an extent.

*Family Disposition to Peritonitis.*—" In company with my friend, Mr. Webb, I visited a young man in 1833, who was labouring under peritonitis; after an illness of ten days he died. A *post-mortem* examination exhibited the peritoneum in a state of high inflammation, covered by copious effusion of puriform lymph, and with which the folds of the intestines were agglutinated together. Two years subsequently to this I again visited, in company with Mr. Webb, a brother of the former, aged 22, under similar circumstances, and with the like result. On the *post-mortem* examination, so like were the appearances that a drawing of the one would most accurately have described the appearances noticed in the other. We were also given to understand that in a different part of the county another brother had died only a few months previously with symptoms exactly similar; no *post-mortem* examination was made. Each of these cases of peritonitis occurred at a time when the disease was by no means prevalent, so that its occurrence must be entirely attributed to family predisposition." 158.

" *Calculi of the Liver.*"—A. B. had long been jaundiced; suffered pain on the right side on pressure, where was evidently a fullness and hardness. He occasionally suffered all the symptoms of the passing of gall-stones; a few days after which there could be discovered in the fæces small dark-coloured matters, not unlike caraway seeds. At the examination after death the gall-bladder was seen involved in a mass of scirrhus. The common biliary duct was scarcely pervious enough to admit an ordinary sized pin. The liver itself was large and hard; internally it was gorged with bile, and here and there could be picked out the small caraway-seed-looking particles. These were, without doubt, moulds of the secreting surface of the liver, and apparently consisted of inspissated bile. They are evidently entitled to be called " calculi of the liver." They are about the tenth of an inch in length, and one-sixteenth in width, slightly curved, and kidney shaped, very light, ten weighing only one grain; their surfaces are covered over with ridge-like reticulations.

*Remedy for the Short-jointed Tape-worm.*—Dr. S. recommends the following formula. ℞. *Cort. radicis punicæ granati* ℥ij. *aquæ* ℔ij., *macera*

*per horas* xxiv., *decoque ad* ℔j., *adde syrupi zingiberis* ℥i. Two ounces of this to be taken every half hour until the worm is expelled. If the head become dizzy, which is not infrequent after the fourth or fifth dose, it should be discontinued. It is quite necessary that the above should be made of the bark of the root, and not of the rind of the fruit; this latter appears to be totally inert as a vermifuge.

### Further Observations on the Variolæ Vaccinæ. By *Robert Ceely*, Esq.

Mr. Ceely has been again investigating this important subject. He has examined into the disease as it appeared in two dairies, and has made a valuable addition to his previous paper. As we gave at the time a full account of *that*, it will be unnecessary to enter into the details of *this*.

In one of the dairies it was thought (and the evidence is circumstantially given) that the vaccine disease originated in the cows in consequence of their exposure to bedding, &c. infected with small-pox. Two of the milkers contracted, from the cows, the variolæ vaccinæ. From one of these, Joseph Brooks, lymph was taken. The following were its effects.

It was directly transferred to three of his younger brothers, a lad older than himself, and two infants. The primary symptoms in his brothers and the young man, aged 19, were manifest on the seventh day, but were mild. After the appearance of the areolæ, the secondary symptoms were promptly excited, and gradually increased till their decline. There was nothing remarkable in the character of the vesicles till the full development of the areolæ, when they became remarkably broad and flat, spreading outwards, turgid with lymph, bursting their walls, and, like those from which they were derived, followed by sloughs and deep slowly healing ulcerations. In the infants, fine and healthy, with thick compact skins, the usual fretfulness and feverishness appeared on the evening of the sixth or seventh day, remitting in the morning and increasing in the evening, in proportion as the areolæ advanced, and declining with them. As usual, nothing remarkable before the eighth day in the appearance of the vesicles: in one the vesicles burst and sloughed; in the other they remained entire, were fine and satisfactory, leaving characteristic crusts, and moderately deep finely reticulated cicatrices.

In the subsequent transfers of the lymph the effects varied according to circumstances. In infants, with tense thick skins, the vesicles, though active, were perfectly free from inconvenience, yielding fine "tamarind stone" crusts and regular scars. On children and adults, where the skin was thin, vascular, or irritable, upon the full expansion of the areolæ, the vesicles spread out broad and flat, and, yielding to the distending influence of their *diluted* contents, burst and terminated in sloughs, more or less deep, followed by a corresponding extent of ulceration; but where the skin was pale and thick, and especially if the patients were also pale and dark in complexion—conditions in every respect most suitable for the use of *new* lymph—then the vesicles were more compact, restricted, bold, and better defined, with proportionately less areola, and often not distinguishable at any period of their course from those induced by ordinary lymph.

Here the crusts were of the ordinary size and form, and the cicatrices of the common depth and extent.

The constitutional symptoms varied not less. In many *infants* the *primary* were scarcely noticed; the *secondary*, proportioned to the texture and vascularity of the parts, the local inflammation, and the temperament of the individual, In *children*, as usual, both were more frequently and more early noticed: the *primary*, on the sixth or seventh day; the *secondary*, at an early period, were marked with vomiting, diarrhœa, much fever, and occasional delirium. In *adults* the same early indications of constitutional symptoms, though not always, were often evinced; and during the *secondary*, as usual, much general complaint was made, some few keeping their beds a day or two. But the primary and secondary constitutional symptoms were comparatively mild in many individuals of the three classes above-mentioned.

At the expiration of three months several patients were tested with variola, by inoculation, with no other than a trifling fugitive inflammation at the point of insertion, or a small vesicle resembling the modified vaccine in form, size, and course, containing a *limpid adhesive* lymph, raised on an indurated base, and terminating on the eighth or ninth day in a small, hard, blackish brown crust, and unattended throughout its course with any constitutional symptoms.

The only *hitch* is the original contamination of the cows. However possible or likely their contamination by small-pox, it cannot be considered as satisfactorily proved.

Mr. Ceely remarks, we think with justice, that the phenomena of the vaccine disease in the cow are worth attentive study. Many country surgeons, were they well acquainted with them, might render an acceptable service to medicine by carefully conducted observations.

The ages of the cows affected were *two* four-years, and *three* seven-years. Of the three milch cows which remained free from the disease *two* were three-years, and *one* probably four-years old. That they really did escape was obvious enough, as far as careful inspections, from time to time, of the teats and udders could enable us to declare; and the total absence of any symptoms of indisposition removed all doubt of the fact. Now these three milch cows were shewn to have been equally exposed to the same primary cause as the others, and there seems to be little doubt that some cows are more susceptible of the vaccine disease than others, while some would appear not to be susceptible at all.

"The phenomena of the disease were well described by the milkers. The primary or incursive symptoms so slight as scarcely to attract notice, as is most commonly the case; though in two instances—one some years since, the other more recently—there was considerable precursory indisposition. The heat and tenderness of the teats, with the presence of *small hard pimples, deep in the skin*, which gradually increased daily, with their inevitable and obvious concomitants, *for more than a week* before 'blistering' or *acumation*, were not less indicative of the vaccine than the correctness of the observers. At this period—the acme—they notice the constitutional symptoms, developed in all their intensity, and describe them with more than ordinary accuracy and precision; they certainly were more clearly marked than usual. Many contingencies however affect the development of the incursive and secondary symptoms; the chief are the season of the year and the actual condition of the animal, and the quantity and quality of food. The retraction and expansion of the cheeks is not always noticed by the careless unobserving milker; nor is the drivelling from the mouth. This last sign was so well marked that the proprietor feared the animals were about to be attacked by the aphtha epizoötica, (which was then prevailing in the adjoining farms,) where it is one of the first signs noticed, though not actually the first in existence, and depends on positive inflammation and vesication of different parts of the mucous membrane of the mouth. In the vaccine disease, however, this is not the case, at least here, where the disease is generally mild; for no vesicles are observed in the mouth, nor difficulty of mastication. The drivelling appears to be the effect of topical irritation in the seat of the disease—the teats and udder; for it is very well known that such a symptom does attend similar degrees of irritation from other causes and in other parts. The secondary constitutional symptoms were noticed in all the patients, including the sturk, and were proportioned in each to the amount of topical irritation. It was remarked, however, that the appetite seemed very little impaired, notwithstanding the wretched appearance of the animals, during the presence of the constitutional symptoms, and the sudden and notable loss of milk." 227.

Mr. Ceely remarks that the ulcerative stage of the variolæ vaccinæ is also a matter deserving careful study, though difficult.

Vaccine ulcers, he says, are generally distinguishable by a rounded elevation, more or less manifest, of their outer margins, and a circumscribed induration of greater or less extent, of their base, with a proportionate depression in their centres of deeper ulceration, sometimes caused by a slough. But these characters are obviously influenced by several circumstances which must be taken into account when attempting our diagnosis; the principal are—stage of the disease, texture of the tissues, site of the ulcers. It is necessary to know that in the early ulcerative stage of the severer forms of some other vesicles nearly as much induration, &c. is occasionally found, especially if the texture of the tissues is lax, and much mechanical irritation has been inflicted. If we can be positively assured that the above-mentioned diagnostic conditions have existed in any given ulcer for three or four weeks, or even longer, especially if it be removed from *severe* casualties, we may fairly presume that it is vaccine; and this presumption will be strengthened, if, under the circumstances, the ulcer be small, for such sized and circumstanced ulcers of most other vesicles, being for the most part superficial and sub-epidemic, speedily heal. But as the texture of the parts in which vaccine vesicles appear varies so much, and as their seat is not uniform in depth, and as elevation of the margin and induration of the base and depression of the centres of the consecutive ulcers are sometimes merely questions of degree, (since time or stage is not always determinable,) it is clear that uncertainty in judging of an individual ulcer must now and then happen. Out of this difficulty we often escape by finding a group of characteristic ulcers on the same animal, or a series of animals, clearly infected one from another. And when we have an opportunity of inspecting ten or twelve, to say nothing of thirty or forty subjects, we seldom fail to detect on some of them the elements of a safe and correct diagnosis, as late as the fifth or sixth week. Such condition of parts very rarely occurs in connection with ulcers resulting from other contagious vesicles, except on peculiar subjects, where the subcutaneous cellular tissue has suppurated and sloughed, and the cicatrizing ulcer has a puckered surface and indurated margin. Most commonly this result is confined to the first subject attacked, which continues also to furnish a succession of milder vesicles for four or five weeks. It must be borne in mind, however, that the vaccine ulcer also is found co-existent with small superficial vesicles or bullæ, both on the teats and the upper, back, and hairy parts of the udder, as in one of the above subjects at Oakley; of the same nature and possessed of the same harmless contents as the vesicles and bullæ which appear (at the acme, or on and after the decline of the vaccine,) on the face, trunk, and limbs of children in high health, and in a few adults with irritable skins.

The Paper is one which highly merits perusal. Before we quit it, we may be allowed to concur in a recommendation of Mr. Ceely's to study Comparative Pathology. It may throw great light on human diseases, and materially improve their treatment.

